# Unloaded Shortening Velocity of Voluntarily and Electrically Activated Human Dorsiflexor Muscles *In Vivo*

**DOI:** 10.1371/journal.pone.0013043

**Published:** 2010-09-27

**Authors:** Kazushige Sasaki, Naokata Ishii

**Affiliations:** Department of Life Sciences, Graduate School of Arts and Sciences, University of Tokyo, Tokyo, Japan; University of Las Palmas de Gran Canaria, Spain

## Abstract

We have previously shown that unloaded shortening velocity (*V*_0_) of human plantar flexors can be determined *in vivo*, by applying the “slack test” to submaximal voluntary contractions (*J Physiol* 567:1047–1056, 2005). In the present study, to investigate the effect of motor unit recruitment pattern on *V*_0_ of human muscle, we modified the slack test and applied this method to both voluntary and electrically elicited contractions of dorsiflexors. A series of quick releases (i.e., rapid ankle joint rotation driven by an electrical dynamometer) was applied to voluntarily activated dorsiflexor muscles at three different contraction intensities (15, 50, and 85% of maximal voluntary contraction; MVC). The quick-release trials were also performed on electrically activated dorsiflexor muscles, in which three stimulus conditions were used: submaximal (equal to 15%MVC) 50-Hz stimulation, supramaximal 50-Hz stimulation, and supramaximal 20-Hz stimulation. Modification of the slack test *in vivo* resulted in good reproducibility of *V*_0_, with an intraclass correlation coefficient of 0.87 (95% confidence interval: 0.68–0.95). Regression analysis showed that *V*_0_ of voluntarily activated dorsiflexor muscles significantly increased with increasing contraction intensity (*R* ^2^ = 0.52, *P*<0.001). By contrast, *V*_0_ of electrically activated dorsiflexor muscles remained unchanged (*R* ^2^<0.001, *P* = 0.98) among three different stimulus conditions showing a large variation of tetanic torque. These results suggest that the recruitment pattern of motor units, which is quite different between voluntary and electrically elicited contractions, plays an important role in determining shortening velocity of human skeletal muscle *in vivo*.

## Introduction

Over the past century, researchers have thoroughly investigated the relationship between the velocity of muscle shortening and the load applied to the muscle (force–velocity relation), where the velocity of shortening decreases with the progressive increase in load. The nonlinear behavior of this relation was first described by Fenn and Marsh [1], and then formulated in terms of a hyperbolic function [2], which enables us to estimate the maximal shortening velocity of muscle (*V*_max_) from the extrapolation of the experimental data. In 1979, Edman [3] developed an alternative approach (referred to as the ‘slack test’) involving multiple quick releases (i.e. rapid shortening) applied to an isometrically contracting muscle fiber, and for the first time measured unloaded shortening velocity (*V*_0_) of single muscle fibers. As with *V*_max_, *V*_0_ is among the most important variables of muscle contractile function, representing the properties of actomyosin kinetics [4], [5]. Strictly speaking, however, there is a difference between *V*_max_ and *V*_0_. In a whole muscle preparation, *V*_0_ is a measure of the shortening velocity of the fastest fibers, whereas *V*_max_ is a function of the force–velocity characteristics of all the fibers [6], [7].

In our previous study [8], we successfully measured *V*_0_ of human plantar flexor muscles in vivo without any confounding effects of series elastic recoil and antagonist coactivation, and demonstrated that *V*_0_, expressed as an angular velocity of ankle joint, increased with muscle activation level defined as a fraction of maximal voluntary contraction torque (MVC). Similar results have been shown in studies investigating *V*_max_ of human wrist flexors [9] and elbow extensors [10]. These results have been interpreted as the manifestation of the ‘size principle’ of motor unit recruitment [11]: the preferential recruitment of small motor units innervating slow-twitch fibers is followed by the progressive recruitment of larger and faster motor units. Nevertheless, some studies have discussed the importance of muscle intrinsic viscosity acting as an internal load in the contractile process, which may also be responsible for the activation-dependent shortening velocity [10], [12]. Given the existence of a fixed internal load within the muscle [13], the shortening velocity will increase with increasing activation, because the load imposed on the muscle decreases, in a relative sense, at high activation level. The lack of experimental data regarding which of these two mechanisms accounts for the activation-dependent shortening velocity requires further investigation, as it is important for both understanding the neuromuscular regulatory mechanisms of human movement and accurate biomechanical modeling of in vivo contractile system.

Recently, artificial electrical stimulation of neuromuscular system has been used not only for experimental purposes, but also for exercise training, physical therapy, and medical rehabilitation. The recruitment order of motor units during electrical stimulation is considered quite different from the natural pattern, which is traditionally interpreted as reversed recruitment order (i.e. from fast to slow type) due mainly to the low input-impedance of fast motor units with thick axons [14]–[16]. However, several researchers have argued that motor unit recruitment during percutaneous electrical stimulation is nonselective, i.e. motor units are driven in a random manner, without obvious sequencing related to contractile properties [17]–[19]. The random recruitment implies the activation of both slow and fast motor units even at relatively low contraction intensity, possibly explaining contradictory evidence as to whether the percutaneous electrical stimulation of human muscles in vivo results in a reversal of the natural recruitment order [15], [20]–[23].

In the present study, we applied the slack test to both voluntary and electrically-elicited contractions of human dorsiflexors. This muscle group was chosen because the tibialis anterior, a major dorsiflexor muscle, lies superficially in the anterior compartment of the lower leg, thus being amenable to stimulation with surface electrodes. The different recruitment pattern of motor units between voluntary and electrically-elicited contractions allowed us to determine the effect of motor unit recruitment on the activation-dependent shortening velocity of human skeletal muscles in vivo. The objective of this study was to test the hypothesis that *V*_0_ of human dorsiflexor muscles increases with contraction intensity during voluntary but not electrically-elicited contractions, because of the differential pattern of motor unit recruitment.

## Methods

### Subjects

Six healthy volunteers (2 women and 4 men) participated in the present study. Their mean (± SD) age, height, and body mass were 19.3±0.9 years, 168.5±7.1 cm, and 58.0±5.6 kg, respectively. All subjects were fully informed of the experimental procedure and purpose of this study, which conformed to the Declaration of Helsinki and were approved by the Ethics Committee for Human Experiments, University of Tokyo. Written informed consent was obtained from each subject prior to participation.

### Equipment

**Figure 1:**
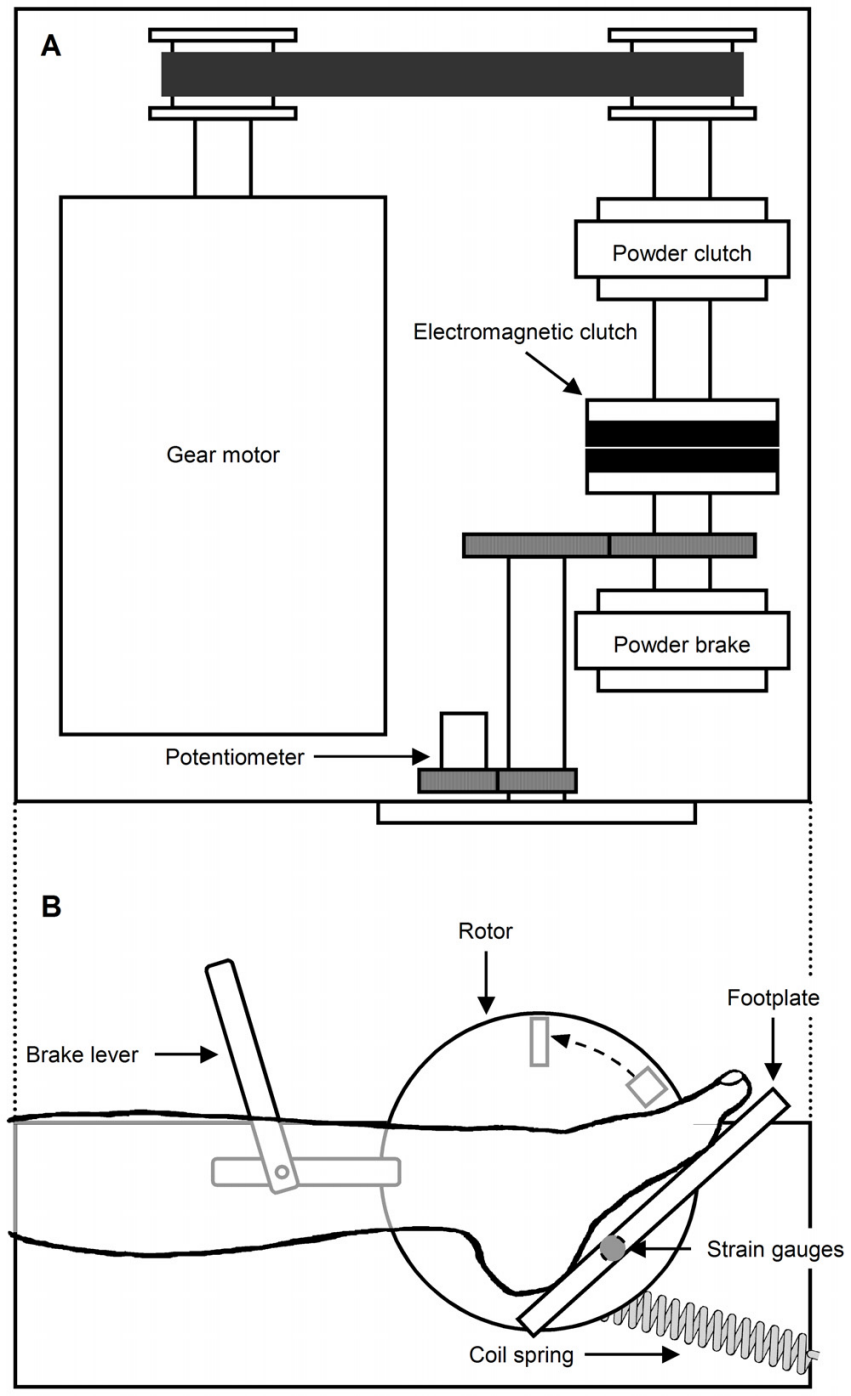
Schematic diagram of motor-driven ankle dynamometer for the slack test. The torque produced by the ankle dorsiflexor as well as plantar flexor muscles was measured with a pair of strain gauges, whereas the ankle rotation was measured with a potentiometer. The torque of a gear motor was transmitted to a footplate via dual clutch system. A quick release was applied to isometrically contracting dorsiflexor muscles by moving a brake lever. A coil spring was attached to the edge of a rotor for increasing initial acceleration of quick-release movement. Two mechanical stops were used to preset the range of angular displacement. For further details, see [8].

To determine *V*_0_ of dorsiflexor muscles, the speed of quick-release movement should be well above the maximal shortening velocity of the muscle. For this purpose, we modified a custom-designed ankle dynamometer driven by a gear motor and dual clutch systems (Fig. 1), which is the same as previously described in detail [8]. A major modification was that a pair of strain gauges, instead of force transducer, was attached to the beam of footplate for measuring the torque produced by the ankle dorsiflexor as well as plantar flexor muscles, and for reducing the weight of footplate. A coil spring was also attached to the edge of rotor for increasing initial acceleration of quick-release movement. These modifications increased the speed of quick-release movement up to 25 rad s^−1^, which is more than three times as high as the estimated maximal velocity of dorsiflexors, 8.1 rad s^−1^ [24].

### Experimental design and setting

We used a within-subject design considering inter-subject variability in *V*_0_ [8]. For each subject, measurements were completed on four different visits. The first visit was used for familiarization with the experimental procedure including measurements of MVC, quick-release trials, and electrical stimulation. In the second visit, the slack test was performed during voluntary contraction to evaluate the reproducibility of measurement (see Statistics and Results). The third and fourth visits involved the slack test during either voluntary or electrically-elicited contraction, the order of which was determined in a counterbalanced fashion. Each visit was separated by at least 4 days.

During the experiment, the subject was seated in a reclining position with the left foot attached firmly to the footplate by using inelastic straps. The lower leg was also immobilized by using inelastic straps. The knee was moderately flexed (approximately 50 deg) to make the subject comfortable and to minimize the contribution of the gastrocnemius muscle, one of the major antagonists, to the ankle joint torque [25]. Both torque and angle signals were sampled at 4000 Hz by using a 16-bit data acquisition system (PowerLab/16SP, ADInstruments, Australia).

### Quick-release experiment during voluntary contraction

MVC of dorsiflexors was measured at an ankle angle of 45 deg of plantar flexion, and was defined as the highest 3-s average in three trials. Following the MVC measurement, *V*_0_ of dorsiflexors was determined by the slack test involving a series of quick-release trials. For each subject, the test consisted of 48 quick-release trials: 8 releases with each of 6 quick-release distances (Δ*L*) expressed in angular units (30, 35, 40, 45, 50, and 55 deg) were given. The subject was instructed to keep ankle joint torque constant with the aid of an oscilloscope (SS-7604, Iwatsu Electric, Japan) before the release, and to keep the level of exertion both during and after the release [8]. The duration of single submaximal voluntary contraction was approximately 3 s, during which the quick release was applied 2 s after the beginning of contraction. The timing of contraction and release was verbally guided by the experimenter. The target-torque levels representing activation level of muscle were set at 15, 50, and 85%MVC. Within each release distance, the trials progressed in the order of 15, 50, and 85%MVC (each repeated twice) to eliminate the possible combined effect of fatigue and post-tetanic potentiation (or myosin light chain phosphorylation) following high-intensity contractions, which could contribute to the inhibition of shortening velocity [26], on the subsequent contractions. The effects of fatigue and potentiation were further minimized by a short (30–60 s) rest between trials as well as a relatively long (approximately 3 min) rest between different release distances. For data correction purposes, an additional release at 0%MVC was given both before and after the set of six trials. The order of release distance was randomized and counterbalanced across subjects.

The smallest release used in this study (30 deg) is more than twice as large as 14 deg, the angular expression of a maximal stretch of series elastic component of human dorsiflexor muscles (estimated from De Zee and Voigt [27], Fig. 3). In addition, the smallest release ended at 15 deg of plantar flexion, where dorsiflexors produced negligible passive torque [28]. Therefore, it is unlikely that the parallel elasticity of dorsiflexors had any effect on the measurement of *V*_0_.

**Figure 2:**
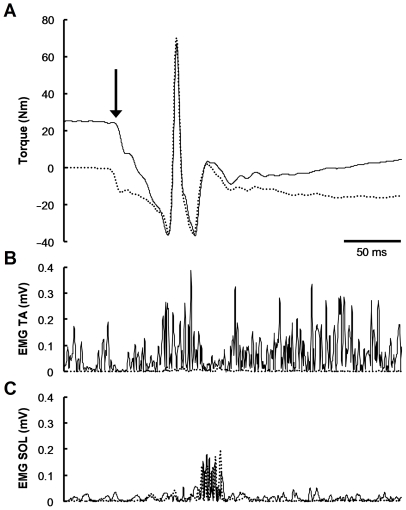
Representative traces of torque (A) and rectified electromyographic activities (EMG) of tibialis anterior (TA, B) and soleus (SOL, C) muscles from quick-release trials (*subject* A). Solid lines indicate the traces from active (50%MVC) release, whereas dotted lines indicate those from passive (0%MVC) release. The vertical arrow indicates the onset of quick-release movement. The large spike in torque signal (A) represents a large deceleration torque due to the mechanical impact at the end of the release. The subsequent ripple mainly reflects the small but very rapid mechanical vibration of the footplate. Approximately 70 ms after the release, there was a decrease in TA activity (B) almost coincided with a burst in SOL activity (C), referred to as unloading and stretch reflexes, respectively. The raw torque signal obtained during passive release was used to correct for the effects of gravity, inertia, and active (stretch reflex) and passive torques of antagonist muscles. Note that the timing and amplitude of stretch reflex of SOL were similar between the active and passive releases.

Previous studies have shown that a certain amount of joint rotation inevitably occurs during ‘isometric’ contraction, due to the compliance of the device and soft tissues [10], [29], [30]. Our preliminary experiment showed that ankle rotation during voluntary isometric dorsiflexion was up to 5 deg, being much smaller than that during voluntary isometric plantar flexion (up to 15 deg). In fact, the effect of ankle joint rotation during isometric contractions can be excluded in our experimental methodology, because the determination of *V*_0_ is based on the differences of Δ*L* obtained from the multiple trials (see Data analysis). No correction of Δ*L* was therefore performed in this study.

### Quick-release experiment during electrically-elicited contraction

Electrical stimulation was applied percutaneously to the common peroneal nerve using an electric stimulator (SEN-7203, Nihon Koden, Japan) with a stimulus-isolation unit (SS104-J, Nihon Koden, Japan). A 2-s tetanic contraction of tibialis anterior muscle was evoked by means of a 0.2-ms rectangular pulse via bipolar surface electrodes. The cathode, an Ag–AgCl ball electrode with a diameter of 10 mm, was placed on the head of the fibula, while the anode, a self-adhesive stimulating electrode (HV-BIGPAD, Omron, Japan), was placed over the mid-belly of tibialis anterior. Supramaximal stimulus intensity was determined at rest by increasing the electrical current until no further increase in twitch torque was observed.

Following the measurement of MVC of dorsiflexor muscles, a series of quick-release trials was performed, in which three stimulus conditions were used in the following order: (1) 50-Hz stimulation with submaximal intensity eliciting a dorsiflexion torque of 15%MVC, (2) 50-Hz stimulation with supramaximal intensity, and (3) 20-Hz stimulation with supramaximal intensity. Essentially, the difference between the first and second conditions corresponds to the difference in the number of activated motor units, whereas that between the second and third conditions corresponds to the difference in the discharge rate of motor units. The quick release was applied 1 s after the onset of electrical stimulation that was verbally indicated by the experimenter. Within each release distance, the trial with each stimulus condition was repeated twice, in which submaximal stimulus condition preceded supramaximal stimulus conditions in order to avoid the possible combined effect of fatigue and potentiation mentioned above.

A total of 48 quick-release trials including releases at 0%MVC were conducted in a manner similar to that during voluntary contraction. To avoid any effect of ‘voluntary’ muscle activity, the subjects were asked to keep their muscles relaxed throughout the trial.

### Electromyography

Electromyographic activities (EMG) of tibialis anterior and soleus muscles were monitored throughout the experiment including the passive (i.e. 0%MVC) release trials. Bipolar Ag–AgCl surface electrodes (10-mm diameter and 25-mm interelectrode distance) were placed on the mid-bellies of these muscles. The reference electrode was placed on the patella. The skin was shaved, cleaned with alcohol, and abraded to reduce the electrode impedance. All EMG signals were differentially amplified (gain 1000×) with an AC amplifier (AB-610J, Nihon Koden, Japan), band-pass filtered (5–1000 Hz), and sampled at 4000 Hz by using the data acquisition system.

### Data analysis

Figure 2 shows representative torque and rectified EMG data during active (50%MVC) and passive (0%MVC) releases. The raw torque signal from the quick-release trials included not only an inertial torque, but also a negative torque due to the stretch reflex of antagonists (e.g. soleus). This negative torque could not be neglected even when the electrical activities of antagonists were small, because the physiological cross-sectional area, a major determinant of force-generating capacity, of plantar flexors was approximately 12 times as large as that of dorsiflexors [31].

**Figure 3:**
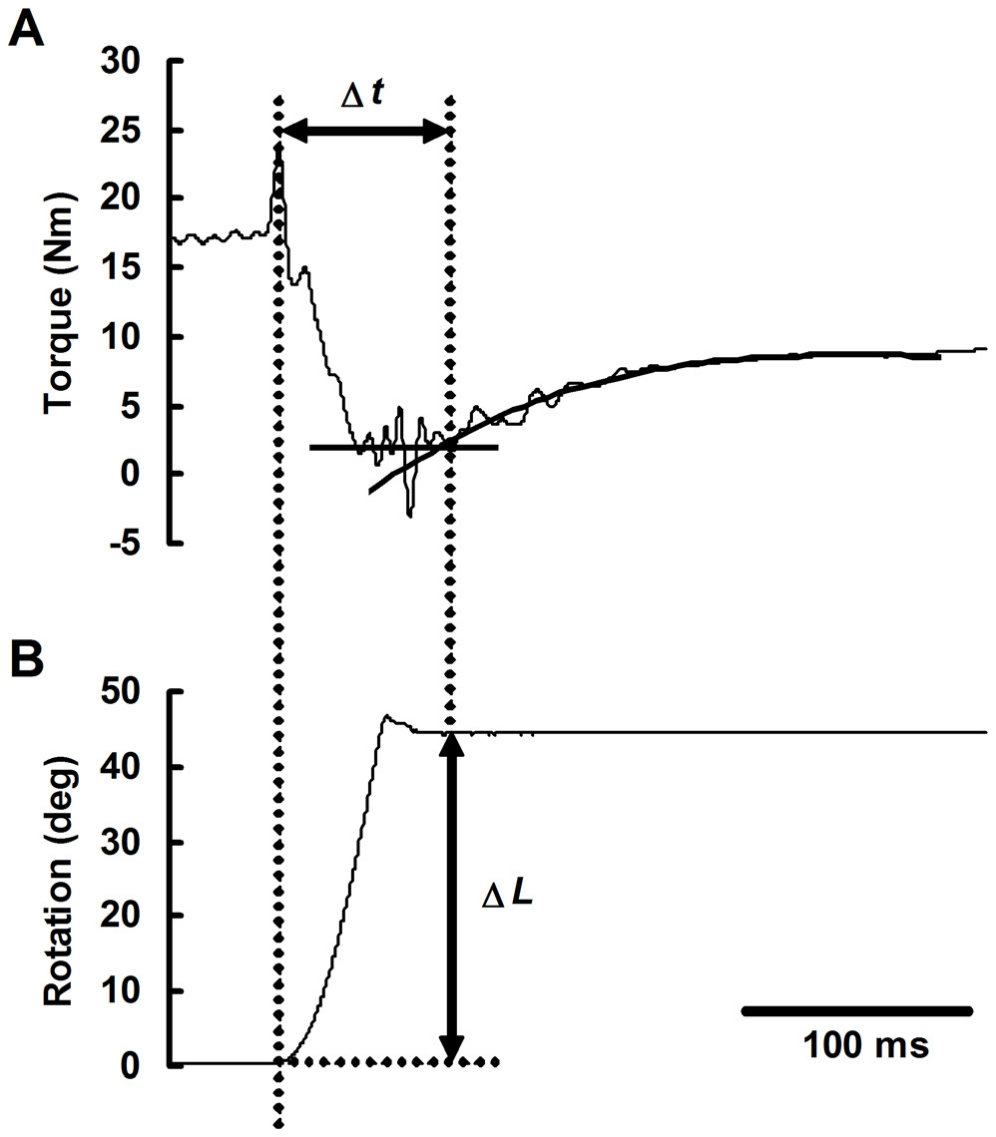
Example of data analysis (*subject* K). From the torque signal corrected for artifacts (A), the time, Δ*t*, was measured as the interval between the onset of release and the beginning of torque redevelopment, the latter of which was defined as the intersection between the torque baseline and the cubic least-squares regression line of the initial portion of torque redevelopment. The quick-release distance (Δ*L*) is expressed as a rotation angle of the footplate (B).

It was found, however, that the peak-to-peak amplitude of stretch reflex of soleus following the quick release of dorsiflexors was independent of contraction intensity including 0%MVC (correlation analysis: *r* = −0.014, *n* = 24, *P* = 0.95). Therefore, the torque due to the stretch reflex of antagonists could be eliminated by simply subtracting the torque signal of passive release from that of active release. This subtraction also corrected for the effects of gravity, inertia, and the parallel elasticity. The corrected torque signal was then smoothed with a zero-lag, fourth-order digital Butterworth filter with a cut-off frequency of 80 Hz.

Figure 3 gives an example of the data analysis. The time, Δ*t*, was measured as the interval between the onset of release and the beginning of torque redevelopment. The former was defined as the point at which the angular displacement exceeded 0.3 deg, whereas the latter was defined as the intersection between the torque baseline and the cubic least-squares regression line of the initial portion of torque redevelopment. Fitting the regression line was less arbitrary than the other methods, such as by-eye estimation [32]. The torque baseline was defined as the 25-ms average of torque signal from the end of the release, during which the muscle was supposed to be taking up the slack. Since two passive release trials were conducted at each release distance, each torque signal from active trials underwent two independent data corrections and analyses, the results of which were averaged for subsequent analysis.

**Figure 4:**
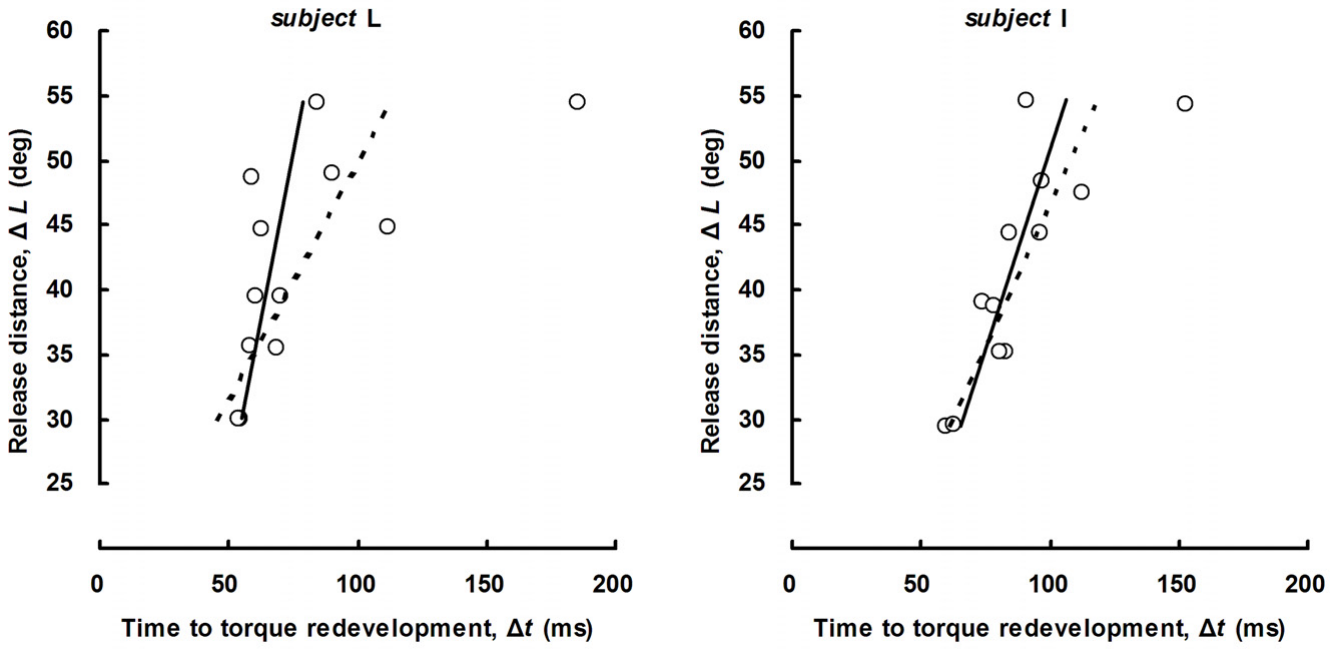
Examples of linear regression lines determined with ordinary least-squares method (dashed line) and iterative reweighted least-squares algorithm (solid line). Representative data from *subjects* L (left panel) and I (right panel) are shown. The iterative reweighted least-squares regression proved to be robust against the outliers due to the fluctuations in torque baseline and the attenuated torque redevelopment.

The value of *V*_0_ has been traditionally defined as the slope of the linear least-squares regression of Δ*t* versus Δ*L*. Statistically, the ordinary least-squares method is so sensitive that a single outlier can considerably change the slope of the regression line. Considering the substantial variability and a limited sample size in the relation between Δ*t* and Δ*L*, as discussed previously [8], we developed an iterative reweighted least-squares algorithm [33] with a quasi-Newton method for the determination of the regression slope (Fig. 4). Briefly, this algorithm minimizes the objective function *S* given by
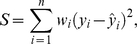
where *n* is the number of data points included in the fit (*n* = 12), *w_i_* is the weight, and *y_i_* and *ŷ_i_* are the measured and predicted values of Δ*t*, respectively. The weight, which depends upon the residual, was determined by using a bisquare weight function with parameters recommended by Holland and Welsch [33]. In order to avoid local minima, several estimated coefficients of slope and intercept, including those obtained with ordinary least-squares regression, were randomly selected as initial values for the iteration. After comparing the results of several calculations, the coefficients minimizing the objective function were used to determine *V*_0_.

### Statistics

Data are presented as means and SD. Statistical analysis was performed with R software version 2.6.1 (http://www.r-project.org/) and Microsoft Office Excel 2003 (Microsoft, USA). The test–retest reliability of the slack test was evaluated in terms of an intraclass correlation coefficient (type 2, 1), coefficient of variation, and Student's paired *t*-test. A linear regression analysis was used to test the dependence of *V*_0_ on contraction intensity. A post-hoc multiple comparison (Student's paired *t*-test with the false discovery rate procedure [34]) was performed, when needed, to compare mean values. *P*<0.05 was considered significant.

## Results

### Test–retest reliability

**Figure 5:**
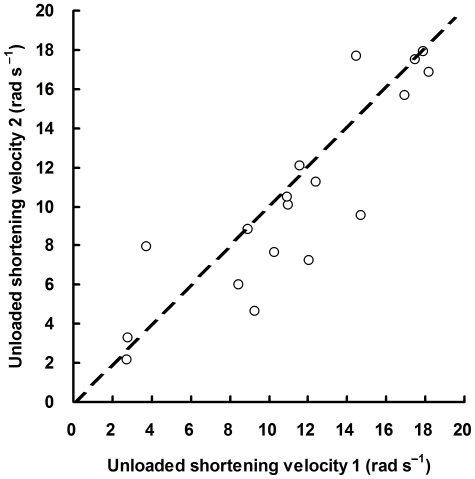
Test–retest reliability of the slack test for human muscle (*n* = 18). Unloaded shortening velocity (*V*_0_) of dorsiflexor muscles was measured twice from 6 subjects at target-torque levels of 15, 50, and 85% of maximal voluntary contraction (MVC). The dashed line represents the line of identity (*y* = *x*).

The test–retest reliability of the slack test during voluntary contraction was evaluated by comparing the values of *V*_0_ obtained from the first and second measurements. As shown in Fig. 5, test–retest data of *V*_0_ measured at 15, 50, and 85%MVC appeared to be evenly distributed around the line of identity (*y* = *x*) with an intraclass correlation coefficient of 0.87 (95% confidence interval: 0.68–0.95). This value was comparable with that of MVC measurements (0.90, 95% confidence interval: 0.53–0.99) obtained from the same subjects. The within-subject coefficient of variation of *V*_0_ for repeated measurements was 11.0% (range: 0.2–36.1%). Student's paired *t*-test revealed no significant difference between the two mean values of *V*_0_ (*P* = 0.13).

### Unloaded shortening velocities of voluntarily and electrically activated muscle

**Figure 6:**
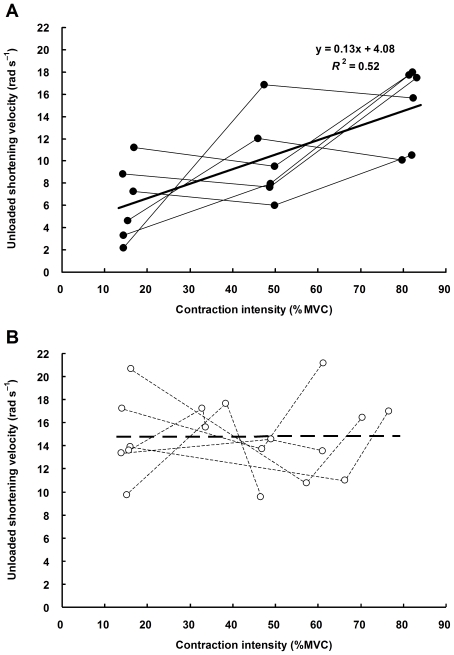
Relations between contraction intensity and unloaded shortening velocity (*V*_0_) of human dorsiflexors during voluntary (A) and electrically-elicited (B) contractions. Shown are individual line plots from 6 subjects. Contraction intensity is expressed as a percentage of maximal voluntary contraction (MVC). *V*_0_ of voluntarily activated muscle significantly increased with increasing contraction intensity (solid line: *R* ^2^ = 0.52, *n* = 18, *P*<0.001), whereas that of electrically activated muscle did not (dashed line: *R* ^2^<0.001, *n* = 18, *P* = 0.98).

Figure 6 shows the relations between contraction intensity (defined as the 500-ms average torque immediately before the release) and *V*_0_ of voluntary (A) and electrically-elicited (B) contractions. The contraction intensity is expressed as a percentage of MVC that was comparable between the two experimental sessions (44.2±9.7 Nm in voluntary contraction session and 44.1±10.0 Nm in electrical stimulation session, *P* = 0.97). The regression analysis showed that *V*_0_ of voluntarily activated muscle significantly increased with contraction intensity (*R* ^2^ = 0.52, *n* = 18, *P*<0.001), whereas that of electrically activated muscle did not (*R* ^2^<0.001, *n* = 18, *P* = 0.98).

Table 1 summarizes the results of multiple comparison procedure. As we expected, the torque output during voluntary contraction matched well the target-torque level, and the dorsiflexion torque induced by the submaximal electrical stimulation corresponded to 15% of MVC. The dorsiflexion torque evoked with supramaximal high-frequency (50 Hz) stimulation was significantly higher than that with low-frequency (20 Hz) stimulation (absolute units: *P* = 0.011; relative units: *P* = 0.005), but smaller than that at 85%MVC (absolute units: *P* = 0.005; relative units: *P* = 0.018). Multiple comparison procedure confirmed the activation-dependent *V*_0_ of voluntarily activated muscle (15%MVC vs. 85%MVC, *P* = 0.005). All the three electrical stimulation protocols resulted in significantly higher values of *V*_0_ (*P*<0.01) than did the lowest voluntary activation (i.e. 15%MVC).

**Table 1:** Contractile parameters in each condition.

	Voluntary contraction			Electrically-elicited contraction		
	15%MVC	50%MVC	85%MVC	SUB50	MAX50	MAX20
**Joint torque (Nm)**	6.7±1.5	21.4±4.6a,b	36.1±7.9a,b,c,d,e	6.6±1.1	25.4±7.5a,b,d	20.9±5.0a,b
**Joint torque (%MVC)**	15.3±1.1	48.4±1.4a,b	81.8±1.0a,b,c,d,e	15.2±0.9	58.2±14.4a,b,d	48.4±11.1a,b
***V*** **_0_ (rad s^−1^)**	6.3±3.2	10.0±3.6	14.9±3.3^a^	14.7±3.4^a^	15.5±3.5^a^	14.2±2.7^a^

Values are means and SD (*n* = 6).

a *P*<0.05 vs. 15%MVC.

b *P*<0.05 vs. SUB50.

c *P*<0.05 vs. 50%MVC.

d *P*<0.05 vs. MAX20.

e *P*<0.05 vs. MAX50.

MVC, maximal voluntary contraction.

SUB50, submaximal 50-Hz stimulation.

MAX50, supramaximal 50-Hz stimulation.

MAX20, supramaximal 20-Hz stimulation.

*V*_0_, unloaded shortening velocity.

## Discussion

The present study was undertaken to investigate whether the differential recruitment pattern of motor units between voluntary and electrically-elicited contractions results in different values of *V*_0_ under various contraction intensities. To our knowledge, studies addressing the effect of motor unit recruitment pattern on muscle force–velocity characteristics have been limited to those using animal models [35], [36], and no studies have so far reported *V*_0_ of human dorsiflexor muscles in vivo. Important findings of this study were: (1) *V*_0_ of voluntarily activated muscle increased with increasing contraction intensity, and (2) *V*_0_ of electrically activated muscle was independent of contraction intensity. A post-hoc power analysis revealed that our experimental design could detect an effect size (*R* ^2^) of 0.38 with a power of 0.8 at a two-sided significance level of 0.05. The statistical power was therefore sufficient for detecting the effect of contraction intensity on *V*_0_ of voluntarily activated muscle (*R* ^2^ = 0.52). On the other hand, no significant effect of contraction intensity on *V*_0_ of electrically activated muscle was mainly due to the near-zero effect size (*R* ^2^<0.001), rather than to the lack of statistical power.

### Methodological consideration

Although the slack test has been widely used and validated in single fiber experiments in vitro, its application to human skeletal muscles in vivo is a great challenge, and needs extensive modification and validation. The special-purpose ankle dynamometer used in this study required only 50 ms even for the largest release (55 deg), which was substantially shorter than the twitch contraction time of dorsiflexor muscles (ranging between 80 and 100 ms in our subjects). As shown in Fig. 2, the quick release during steady-state isometric contraction caused a rapid drop in joint torque while the agonist tibialis anterior muscle remained activated. Approximately 70 ms after the release, there was a burst in the antagonist muscle activity (stretch reflex), which later emerged as a torque output because of electromechanical delay in the neuromuscular system. Since the timing and amplitude of the antagonist stretch reflex were almost independent of agonist activity (see Fig. 2), fluctuations in torque output due to the antagonist muscle activity could be excluded by subtracting the torque record of passive release from that of active release. Concurrently with the stretch reflex, there was a decrease in the activity of tibialis anterior muscle, referred to as the ‘unloading reflex’ [37], especially when the contraction intensity was high. Although these reflexes were partly responsible for the variations in Δ*t*, the slack test has a notable methodological advantage in that the differences, not the absolute values, of Δ*t* from the multiple trials are used for the calculation of *V*_0_, minimizing any systematic errors due to the muscle reflex.

It is likely that the test–retest reliability of *V*_0_ is highly dependent on the number of data points used for determining the slope of Δ*L*–Δ*t* relation. The number of quick-release trials, however, should be minimized to reduce the strain on the subjects. To reconcile these conflicting requirements, we used the iterative reweighted least-squares algorithm for the robust determination of *V*_0_.

As a result of several modifications noted above, we obtained acceptable reproducibility of *V*_0_. Although the average coefficient of variation was relatively high (11.0%), and the reproducibility was examined only in 6 subjects, the intraclass correlation coefficient for repeated measurements of *V*_0_ (0.87) was similar to that for repeated measurements of MVC (0.90) in the same subjects. This result indicates that the determination of human muscle *V*_0_ by using the slack test is as reproducible as the muscle strength measurement that has been commonly used in the area of human muscle physiology. It should be noted, however, that the reproducibility of MVC was influenced, to some extent, by the motor learning effect [38].

### Unloaded shortening during voluntary contraction

As with plantar flexors in our previous study [8], *V*_0_ of voluntarily activated dorsiflexor muscles significantly increased with increasing contraction intensity (Fig. 6A). The values of *V*_0_ ranged from 6.3±3.2 rad s^−1^ at 15%MVC to 14.9±3.3 rad s^−1^ at 85%MVC. A direct comparison of these values with those of plantar flexors (e.g. 8.6±2.6 rad s^−1^ at 60%MVC) determined in our previous study [8] seems difficult, because of the differences in subjects, experimental protocols, and data analyses. Nevertheless, relatively high *V*_0_ of dorsiflexors may be explained by the difference in muscle architectural characteristics between dorsiflexors and plantar flexors. Human tibialis anterior muscle, a major dorsiflexor, is characterized as its short moment-arm length [39] and long fascicle length [40], both of which would be advantageous for achieving high angular velocity of ankle dorsiflexion. From the literature noted above [39], [40], we can assume that tibialis anterior muscle has an average moment-arm length of 40 mm and fiber (fascicle) slack length (*L*_0_) of 60 mm. Given a negligible effect of pennation angle for simplicity, *V*_0_ of tibialis anterior muscle may vary from 4.2 *L*_0_ s^−1^ at 15%MVC to 9.9 *L*_0_ s^−1^ at 85%MVC. These values are somewhat smaller than *V*_max_ of intact, slow-twitch soleus (7.02 *L*_0_ s^−1^) and fast-twitch extensor digitorum longus (13.00 *L*_0_ s^−1^) muscles in rats, respectively, measured at 35°C [41]. The differences can be explained by the scaling effect, i.e. the significant negative relationship between body size and *V*_0_ in muscle fibers expressing a given myosin isoform [42], [43]. By contrast, these values are considerably higher than *V*_0_ of human skinned fibers measured at 12–15°C (slow-twitch fibers: 0.26–1.45 *L*_0_ s^−1^; fast-twitch fibers: 1.12–4.33 *L*_0_ s^−1^ [44]–[46]), probably due to the high temperature sensitivity of *V*_0_ [3], [44]. Besides the difference in temperature, the experimental conditions under which *V*_0_ of skinned fibers was measured were far from the physiological conditions in vivo.

The mean value of *V*_0_ at 15%MVC was significantly lower than that at 85%MVC (Table 1). Since the slow-twitch fibers account for approximately 70% of total fiber number in the human tibialis anterior muscle [47], [48], it is reasonable to assume that at 15%MVC, only the small motor units innervating slow-twitch fibers are activated. It is also a reasonable assumption that the remaining fast-twitch fibers are progressively recruited with increasing contraction intensity until near maximal activation. In fact, the additional recruitment of motor units was observed up to 70%MVC in the human tibialis anterior muscle [49]. The additional, progressive recruitment of larger motor units innervating faster fibers should result in the progressive increase in *V*_0_ of dorsiflexor muscle, because among the activated fibers, the fastest ones are considered the major determinant of *V*_0_ at the whole muscle level [6], [7].

### Unloaded shortening during electrically-elicited contraction

Although the use of three stimulus conditions resulted in a large variation of tetanic torque (Table 1), the linear regression analysis revealed that *V*_0_ of electrically activated dorsiflexor muscles was independent of contraction intensity (Fig. 6B). The similarity in *V*_0_ between the second (supramaximal 50-Hz stimulation) and third (supramaximal 20-Hz stimulation) conditions is not surprising, because supramaximal stimulation activates the largest possible muscle mass for a given electrode location [50], most likely including the fastest motor units within the muscle. On the other hand, the difference between the submaximal and supramaximal stimulus conditions essentially corresponds to the difference in the number of activated motor units. Contrary to the voluntary contraction, artificial activation of different number of motor units resulted in similar *V*_0_. The result suggests that the number of activated motor units is not necessarily a determinant of *V*_0_ in human muscle contractions in vivo.

During electrical stimulation with surface electrodes, superficial tibialis anterior muscle, which accounts for approximately 60% of physiological cross-sectional area of dorsiflexors [31], was supposed to be the only muscle activated. The tetanic torque evoked with supramaximal 50-Hz stimulation (58.2±14.4% of MVC) was thus reasonable, and consistent with the data of previous studies on electrically-stimulated human tibialis anterior muscle [27], [40]. During voluntary contraction, on the other hand, all dorsiflexor muscles such as tibialis anterior, extensor digitorum longus, and extensor hallucis longus, would simultaneously produce the dorsiflexion torque. Therefore, one might argue that, during electrically-elicited contraction, the activity of tibialis anterior should be higher than that during voluntary contraction, even though the resultant dorsiflexion torque was the same. Assuming a selective stimulation of tibialis anterior accounting for 60% of force-generating capacity of dorsiflexors, a quarter of its maximal activation is required to produce dorsiflexion torque corresponding to 15%MVC. Such a 10% difference in the activity of tibialis anterior muscle may be partly responsible for the difference in *V*_0_ between voluntary and electrically-elicited contractions of equal intensity (15%MVC). However, even if the above assumptions are correct, the comparable *V*_0_ among different stimulation intensities cannot be fully explained, because the estimated activity of 25% is still far from the maximal activation attained with supramaximal stimulation. In addition, participation of antagonists during voluntary contraction would also result in the increase in the activity of tibialis anterior, because in this study, contraction intensity was calculated from the net ankle joint torque reflecting both the agonist and antagonist muscle activities. Therefore, the difference in the activity of tibialis anterior muscle between submaximal voluntary and electrically-elicited contractions would in reality be smaller than 10%.

In addition to the selective recruitment of superficial muscle, the spatially-fixed activation of motor units within the muscle is also considered an important feature of percutaneous electrical stimulation [17], [19]. We cannot exclude the possibility that the nonuniform motor unit activation within the muscle contributes to the intra- and inter-subject variations in *V*_0_ of electrically activated muscle observed in the present study. In most of human muscles, however, the slow-twitch and fast-twitch fibers are not differentially expressed, but randomly arranged in a mosaic pattern [47], in which case the stimulation of different portion of muscle should end up with similar *V*_0_.

Although a great deal of research has focused on the difference in motor unit recruitment between voluntary and electrically-elicited contractions, the precise recruitment order of motor units during percutaneous electrical stimulation of human muscle remains unresolved (for reviews, see [16], [17], [19]). The preferential or selective activation of large motor units innervating fast-twitch fibers is the traditional interpretation [14], [16], whereas the nonselective or random recruitment of motor units is a recently proposed concept [17]–[19]. The similarity in *V*_0_ of submaximally and supramaximally stimulated dorsiflexor muscles seems consistent with both possibilities, implying not only supramaximal but also submaximal stimulation can activate the fastest motor units even when the contraction intensity is low. Although it is beyond the scope of this study to discuss which of the above two possibilities is more likely, a full understanding of how the various types of motor units are driven during percutaneous electrical stimulation of human muscle is important from a practical point of view, requiring further investigation.
